# TRAIL combinations: The new ‘trail’ for cancer therapy (Review)

**DOI:** 10.3892/ol.2014.1922

**Published:** 2014-02-27

**Authors:** ALAA REFAAT, AHMED ABD-RABOU, ASMAA REDA

**Affiliations:** Center for Aging and Associated Diseases, Zewail City of Science and Technology, Giza 12588, Egypt

**Keywords:** tumor necrosis factor related apoptosis-inducing ligand, death receptor 5, apoptosis, cancer therapy

## Abstract

Tumor necrosis factor-related apoptosis-inducing ligand (TRAIL) therapy is anticipated to be one of the most effective cancer treatments. However, resistance to TRAIL therapy remains a challenge facing the development of anticancer strategies. To circumvent this problem, TRAIL combinations have been experimented with for over ten years to induce synergism or sensitize resistant cancer cells. By analyzing the signaling pathways triggered by these combinations, this review has defined a set of core targets for novel combinatorial treatments. The review suggests specific pathways to be targeted together with TRAIL for more efficient treatment, including cellular FLICE inhibitory protein and its downstream survival factors, the Bcl-2 family and other prominent targets. The suggested pathways provide new avenues for more effective TRAIL-based cancer therapy.

## 1. Introduction

Programmed cell death is considered a defensive mechanism to eliminate harmful and defective cells. Disturbances in the signaling pathways involved in programmed cell death may lead to uncontrolled cell proliferation and eventually cancer. Therefore, recent studies have focused on apoptosis, autophagy and necroptosis as strategic targets for novel cancer therapies (1). Apoptosis is of particular importance due to its pivotal role in controlling irregular cell proliferation through its well-defined mechanism. Apoptosis can be either initiated by ligands that bind to receptors on the cell membrane (extrinsic pathway) or initiated from intracellular signals (intrinsic or mitochondrial pathway) (2). With regard to ligand-induced apoptosis, characterized ligands and corresponding death receptors include Fas ligand/Fas receptor, tumor necrosis factor (TNF) α/TNF receptor 1, Apo-3 ligand/death receptor (DR) 3, TNF-related apoptosis-inducing ligand (TRAIL)/DR4 and TRAIL/DR5 (2).

TRAIL was first characterized in the 1990s by Wiley *et al* (3). Its potential use in cancer treatment was described later (4). TRAIL is characterized by its ability to selectively induce apoptosis in tumor cells but not in normal cells, qualifying as a potential drug specific for different types of cancer, including breast, bladder, lung and liver (5–9). TRAIL is a cytokine secreted by the majority of normal tissues as a part of the natural immune reaction. It has been demonstrated that breast-feeding women produce high levels of TRAIL in their milk, which may contribute to anticancer effects in infants (10). Collectively, TRAIL plays a significant role in cancer eradication and the prevention of proliferation, while being less likely to cause chemotherapeutic toxicity than established treatments (11). The growing interest in TRAIL-based interventions has led to the development of recombinant human TRAIL (rhTRAIL) as a promising therapy for different types of human cancer (12).

This review will summarize the apoptotic pathway of TRAIL monotherapy in cancer cells, and how resistance develops against it. Subsequently the outcome of studies that have used TRAIL as a part of anticancer combinatorial therapy will be summarized and a set of targets that can be subsequently targeted specifically in combination with rhTRAIL to efficiently eliminate cancer will be identified.

## 2. Signaling pathway of TRAIL

**Figure 1:**
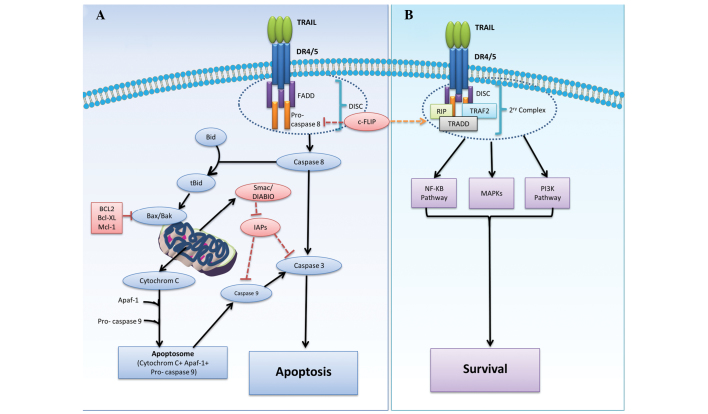
Dual opposing signaling pathways of TRAIL. (A) The apoptotic signaling pathway. (B) The resistance pathway developed against TRAIL-induced apoptosis. TRAIL, tumor necrosis factor-related apoptosis-inducing ligand.

In addition to binding to DR4 and DR5, TRAIL can bind decoy receptor (DcR) 1, DcR2 and the soluble receptor osteoprotegerin. However, only DR4 and DR5 can produce apoptotic signals through their intracellular death domain (13). As illustrated in Fig. 1A, the apoptotic signaling pathway of TRAIL is triggered by binding of trimerized TRAIL to DR4 and/or DR5, followed by receptor clustering leading to the recruitment of Fas-associated protein with death domain (FADD). FADD adaptor protein then recruits pro-caspase 8, forming the death-inducing signaling complex (DISC) known as the primary complex. The recruitment of pro-caspase 8 causes activation of DISC and the subsequent cleavage of caspases 3, 6 and 7, resulting in membrane blebbing, DNA fragmentation and nuclear shrinkage. In certain cases, activated caspase 8 requires the engagement of a mitochondrial response in what is known as the intrinsic pathway. In the intrinsic pathway, active caspase 8 cleaves the BH3-interacting domain death agonist (Bid) to truncated Bid (tBid). tBid then binds Bcl-2-associated X protein (Bax) and Bcl-2 homologous antagonist killer (Bak), then translocates to the mitochondria. This results in a change in mitochondrial membrane polarization and the release of mitochondria-derived activator of caspase (Smac) (14). tBid also induces mitochondrial release of cytochrome *c* (15), which conjugates with ATP and apoptotic peptidase activating factor 1 (Apaf-1) to form a structure known as the apoptosome. This apoptosome is essential for the activation of caspase 9 and eventual activation of caspases 3, 6 and 7 (2,13,16).

## 3. Resistance developed against TRAIL-induced apoptosis

Current TRAIL-induced apoptosis strategies are hampered by the scarcity of death receptors expressed on the cell surface, and thus the inefficient targeting of these cells by TRAIL/agonistic monoclonal antibody (mAb). In addition, development of resistance to rhTRAIL/agonistic mAb has unfavorable negative implications for such therapies (16). Although DISC is considered a critical step in the initiation of apoptotic signaling through the activation of pro-caspase 8, cellular FLICE inhibitory protein (c-FLIP), which shares sequence homology with caspase 8, may inhibit caspase activation by competing for FADD binding, as illustrated in Fig. 1B. In the presence of c-FLIP, FADD and pro-caspase 8, together with receptor-interacting protein (RIP), TNF receptor-associated factor 2 (TRAF2), IκB kinase and TNFR1-associated death domain (TRADD), form a secondary complex responsible for the activation of non-apoptotic signals initiated through the phosphoinositide 3-kinase (PI3K)/Akt, nuclear factor κB and mitogen-activated protein kinase (MAPK) pathways. However, a previous report refers to c-FLIP as a pro-apoptotic protein and therefore the survival process may require further clarification (17).

Another group of molecules involved in the resistance mechanism is the inhibitor of apoptosis (IAP) family, which includes X-linked IAP, cellular IAP (c-IAP) 1, c-IAP2 and survivin. This group of molecules can inhibit the activity of caspases 3, 7 and/or 9. Nevertheless, this effect can be antagonized by Smac/direct inhibitor of apoptosis binding protein with low pi (DIABLO), which is released from mitochondria during apoptosis (18).

## 4. Signaling pathway of TRAIL combinations

Facing acquired resistance to TRAIL-targeted cell death, an alternative approach has been utilized through which TRAIL is combined with other drugs that can be more effective than a single therapy. The major objective of combinatorial TRAIL is to either synergize the activity of TRAIL or to sensitize TRAIL-resistant cells. Previous studies by the authors demonstrated that several natural compounds, including curcumin, cinobufotalin and berberine may be used solely or in combination to treat various disorders, including cancer (19–22). To that end, natural compounds are involved in the majority of combinatorial strategies directed towards synergizing TRAIL and/or sensitizing resistant cancers to TRAIL.

**Figure 2:**
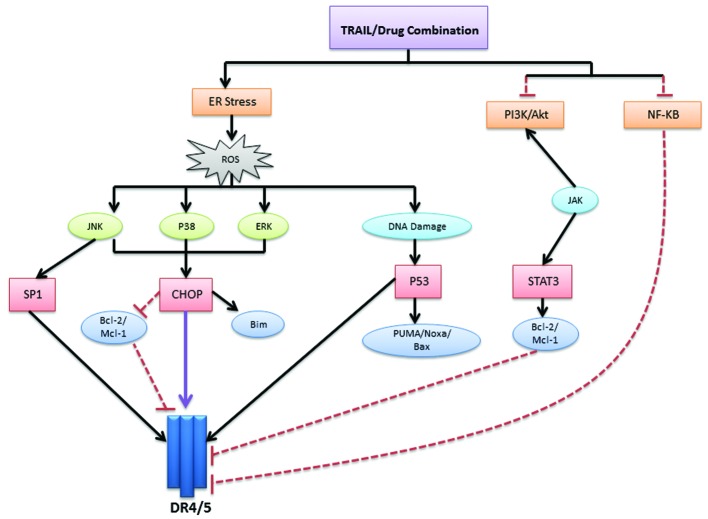
Signaling pathway of TRAIL/drug combinations. TRAIL, tumor necrosis factor-related apoptosis-inducing ligand.

Combinatorial strategies mainly initiate their action through endoplasmic reticulum (ER) stress, resulting in the upregulation of DR5 and/or DR4 followed by increased TRAIL-induced apoptosis (23–25) (Fig. 2). ER stress primarily causes the release of reactive oxygen species (ROS) (26,27), which is considered a central checkpoint from which several signaling pathways can be triggered. Another downstream checkpoint is the activation of CCAAT-enhancer-binding protein homologous protein (CHOP) via p38/extracellular-signal-regulated kinase (ERK) MAPKs, which in turn increase the transcription of DR5 (28,29), enhance pro-apoptotic proteins (such as Bim) (30) or downregulate the Bcl-2 and Mcl-1 survival proteins (29,31). The third member of the MAPK family, the c-Jun N-terminal kinases (JNKs), can also upregulate DR5 (via an Sp1-mediated mechanism) and downregulate Bcl-2 and Mcl-1 (32). ROS may also cause DNA damage and p53 activation, leading to direct DR5 upregulation (the extrinsic apoptotic pathway) (33,34) or activation of p53 upregulated modulator of apoptosis (PUMA), phorbol-12-myristate-13-acetate-induced protein 1 (Noxa) and Bax pro-apoptotic proteins (the intrinsic apoptotic pathway) (35,36).

In addition to ER stress, TRAIL combinations can act by downregulating NFκB, PI3K/Akt or Janus kinase (JAK)/signal transducer and activator of transcription (STAT) pathways. Previous studies have also revealed that the downregulation of c-FLIP appears to be an important mechanism for improved apoptotic response (37).

## 5. Impact of current TRAIL combinations on future therapeutic strategies

The remainder of this review focuses on candidates that can be targeted in combination with TRAIL as a part of emerging treatments for unresponsive cancer.

### C-FLIP and downstream survival factors

**Figure 3:**
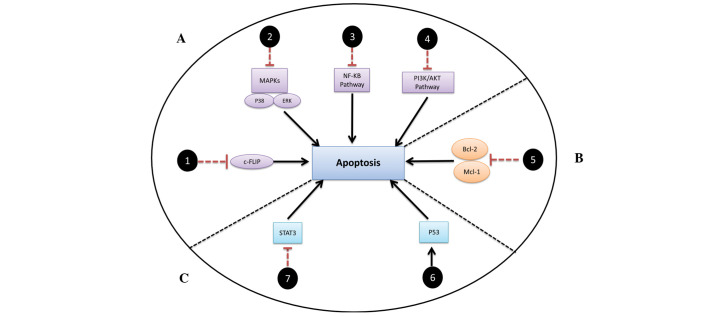
Schematic diagram showing possible targets as part of future tumor necrosis factor-related apoptosis-inducing ligand-based therapies enhancing apoptosis. (A) Targeting C FLIP and downstream survival factors. (B) Targeting the Bcl 2 family. (C) manipulating p53 and STAT3.

C-FLIP has been consistently reported to have a role in conferring resistance through shifting the TRAIL-mediated apoptotic pathway towards secondary complex formation (*vide supra*). The secondary complex triggers the initiation of certain survival pathways, including NFκB and PI3K/Akt, which may promote resistance. Treatment of TRAIL-resistant cancer cells with chemotherapeutic agents, including camptothecin, celecoxib and cisplatin, results in the downregulation of c-FLIP and thus sensitizes the resistant cancer cells to TRAIL (38). Thus, the inhibition of c-FLIP would be of great value in sensitizing cancer to TRAIL by inhibiting the formation of the secondary complex (37) (Fig. 3A, track 1).

The MAPK family includes three pathways: ERK, JNK and p38. Whereas ERK is associated with cell survival and proliferation, JNK is a promoter of cell death and apoptosis (39). Notably, targeting ERK in non-tumor cells has been shown to induce resistance against TRAIL, implying that an ERK inhibition/TRAIL combination would efficiently target tumor cells without harming normal cells (40). The role of p38 depends on the upstream activation and the type of stimuli (41). Previous studies have reported that p38 has a role in tumor growth and cell survival through control of a signaling network responsible for cell proliferation (20,42). Together, these findings may lead to systematic targeting that specifically inhibits ERK or p38 in combination with rhTRAIL. However, pharmacological parameters should be optimized to avoid the loss of CHOP/ERK− and/or p38-mediated apoptosis due to the upregulation of DR4/DR5 (Fig. 3A, track 2).

NFκB, which has been found to be downregulated by TRAIL combinations, is an important candidate for new targeted inhibitors due to its pivotal survival roles (43). The inhibition of the NFκB pathway with TRAIL therapies may serve as a solution for unresponsive or resistant tumors (Fig. 3, track 3).

Finally, the deregulation of the PI3K/Akt pathway has been observed in several types of human cancer (44,45). This enhances the survival of cancer cells by promoting cell cycle progression, proliferation, invasion and angiogenesis (46–48). Activation of this pathway is correlated with the incidence of high-grade tumors and a decrease in apoptosis (49). It has been reported that the inhibition of PI3K leads to synergistic effects in TRAIL-induced apoptosis (50). Therefore, the use of PI3K-specific inhibitors (such as LY294002 and Wortmannin) may have a significant therapeutic outcome when combined with rhTRAIL (Fig. 3A, track 4).

### Bcl-2 family

Bcl-2 family proteins play the main role in the regulation of apoptosis. They are divided into anti-apoptotic and pro-apoptotic proteins (51,52). Upregulation of anti-apoptotic proteins, including Bcl-2 and Mcl-1, or downregulation of pro-apoptotic Bax and Bak has been associated with resistance to TRAIL and recurrence of cancer (53,54). It appears that the ratio of pro- versus anti-apoptotic Bcl-2 proteins is crucial in regulating the susceptibility of cancer cells to apoptosis. Shifting this balance towards apoptosis provides a viable tool in initiation of cancer cell death (55). Thus, searching for novel strategies to enhance TRAIL concurrent with anti-apoptotic protein inhibition would be of significant therapeutic benefit (56,57). Combining Bcl-2-specific inhibitors (such as ABT-737 and HA14-1) with TRAIL would be a powerful strategy against cancer (56,58). In addition, Bcl-2- or Mcl-1-specific knockdown alongside TRAIL therapy would have potential for inducing apoptosis (Fig. 3B, track 5).

### Others

TP53 (*p53*) is considered one of the four major tumor suppressor genes together with phosphatase and tensin homolog, alternate reading frame and inhibitor of cyclin-dependent kinase 4a. The main function of p53 is cancer prevention through controlling cell death pathways. In addition, it negatively regulates the transcription of important anti-apoptotic genes including Mcl-1, Bcl-2 and survivin (59). Several reports, including a recent study by the authors (20) have shown p53 mutation to be a hallmark of TRAIL resistance *in vitro*. A critical factor for the TRAIL resistance of p53-mutant cell lines is the limited upregulation of the expression of DR4 and DR5 by mutant p53 (34,60–62). Previous studies have investigated the modulation of p53 using small molecules that restore p53 function in tumor cells. p53 reactivation and induction of massive apoptosis (PRIMA-1) and mutant p53-dependent induction of rapid apoptosis are two examples of this new class of compound which exhibits efficacy in killing tumor cells that express mutant p53 (63). In particular, PRIMA-1 has been investigated *in vitro*, *in vivo* and is currently in clinical trials (63). Elucidating the mechanism of action of this class and combining it with other anti-neoplastic agents is therefore becoming increasingly important. Selective restoration of mutant p53 to sensitize TRAIL-resistant cells to rhTRAIL via the upregulation of DR4/DR5 is thus a promising therapeutic strategy (Fig. 3C, track 6).

Studies have revealed that STAT3 is negatively regulated in response to TRAIL combinations, which eventually leads to the upregulation of DRs via the manipulation of anti-apoptotic proteins (64,65). It is therefore suggested that specific inhibition of STAT3 (by Stattic, for example) would lead to induction of apoptosis (Fig. 3C, track 7).

## 6. Conclusion

This review has summarized the outcome of various studies carried out during the past fifteen years and the role of TRAIL combinations in enhancing apoptotic signaling pathways. It has highlighted the pathways activated or downregulated by those combinations which enhance apoptotic cell death and eliminate resistance to single TRAIL therapy. Future therapeutic strategies should capitalize on selective modulators that regulate those pathways as a part of a combined TRAIL therapy. In addition, the study has outlined several promising targets for direct intervention together with rhTRAIL therapy. It remains to be verified whether these new combinations are effective therapies.
